# Numerical Simulation of Electromagnetic Nondestructive Testing Technology for Elasto–Plastic Deformation of Ferromagnetic Materials Based on Magneto–Mechanical Coupling Effect

**DOI:** 10.3390/s24227103

**Published:** 2024-11-05

**Authors:** Xiangyi Hu, Xiaoqiang Wang, Haichao Cai, Xiaokang Yang, Sanfei Pan, Yafeng Yang, Hao Tan, Jianhua Zhang

**Affiliations:** 1 School of Mechatronics Engineering, Henan University of Science and Technology, Luoyang 471003, China; wang_xq2002@163.com (X.W.); chc1226@haust.edu.cn (H.C.); yangxiaokang@haust.edu.cn (X.Y.); 2 School of Vehicle and Traffic Engineering, Henan University of Science and Technology, Luoyang 471003, China; pansaifei2023@haust.edu.cn (S.P.); 9906477@haust.edu.cn (Y.Y.); 3 Modo Institute of Technology, International Education College, Henan University of Science and Technology, Luoyang 471000, China; 13598217487@163.com; 4 Key Laboratory of High Efficiency and Clean Mechanical Manufacture, Ministry of Education, National Demonstration Center for Experimental Mechanical Engineering Education, School of Mechanical Engineering, Shandong University, Jinan 250061, China; jhzhang@sdu.edu.cn

**Keywords:** magneto–mechanical effect, electromagnetic nondestructive testing technology, elasto–plastic deformation, ferromagnetic materials, numerical simulation

## Abstract

A numerical tool for simulating the detection signals of electromagnetic nondestructive testing technology (ENDT) is of great significance for studying detection mechanisms and improving detection efficiency. However, the quantitative analysis methods for ENDT have not yet been sufficiently studied due to the absence of an effective constitutive model. This paper proposed a new magneto–mechanical model that can reflect the dependence of relative permeability on elasto–plastic deformation and proposed a finite element–infinite element coupling method that can replace the traditional finite element truncation boundary. The validity of the finite element–infinite element coupling method is verified by the experimental result of testing electromagnetic analysis methods using TEAM Problem 7. Then, the reliability and accuracy of the proposed model are verified by comparing the simulation results under elasto–plastic deformation with experimental results. This paper also investigates the effect of elasto–plastic deformation on the transient magnetic flux signal, a quantitative hyperbolic tangent model between B*z*_pp_ (peak–peak value of the normal component of magnetic flux signal) and elastic stress, and the exponential function relationship between B*z*_pp_ and plastic deformation is established. In addition, the difference and mechanism of a magnetic flux signal under elasto–plastic deformations are analyzed. The results reveal that the variation of the transient magnetic flux signal is mainly due to domain wall pinning, which is significantly affected by elasto–plastic deformation. The results of this paper are important for improving the accuracy of quantitative ENDT for elasto–plastic deformation.

## 1. Introduction

Electromagnetic nondestructive testing technology (ENDT) has been widely used in modern industries, such as oil–gas pipelines, high-speed railways, bridges, cross-space steel structures, aerospace, and nuclear equipment, for its excellent properties of fast detection speed, high efficiency, and high precision [1,2,3,4]. The principle of ENDT is the magneto–mechanical effect, which can be described as when a magnetic field is applied to a specimen, the elasto–plastic stress or defects in a ferromagnetic material will change the distribution of the magnetic field [3,4]. Theoretical models based on the magneto–mechanical coupling effect have always been a research hotspot in magnetic nondestructive testing. The numerical simulation methods combining theoretical models with numerical analysis methods can provide a basis for studying the detection mechanism and improving the detection efficiency of ENDT. The accuracy of the numerical simulation results is the basis for ensuring the accuracy of quantification and inversion of ENDT. Therefore, it is of great significance to research a high-precision numerical analysis method that includes magneto–mechanical model and simulation methods and establish the relationship between magnetic signal and elasto–plastic deformation of ferromagnetic materials for the development of quantitative ENDT.

In recent decades, various magneto–mechanical models have been extensively considered. The Zheng Xiao-Jing Liu Xin-En model (Z-L model) [5], based on thermodynamic theory and magnetic domain motion theory, is widely used because of its obvious physical significance. Based on the Z-L model [5], Shi [6] introduced a new concise magnetostrictive strain expression, which greatly promoted the application of the magneto–mechanical theoretical models. To describe the magneto–mechanical effect of ferromagnetic materials under weak magnetic fields, Sun et al. [7] proposed a new stress-induced magnetic model based on the classical Z-L model, which can effectively describe the dependence of the relative permeability of materials on elastic stress in different directions. In addition, based on the law of energy conservation, Wang et al. [8] established a magneto–mechanical model between the relative permeability and stress of ferromagnetic materials, which is widely used in magnetic nondestructive testing. In further analysis, Shi et al. [2,9] compared the theoretical results of the relative permeability model based on Wang’s model [8] with their proposed model and found that Wang’s model [8] could not reflect the variation of magnetic signal with stress. Compared with Wang’s model [8], the theoretical results of Shi’s [2,9] model are more consistent with the experimental results. Moreover, to describe the magnetic signal caused by plastic deformation, Wang et al. [10] proposed a plastic deformation-induced magnetic field model, which could reflect the effect of plastic deformations on magnetization, but Wang’s model [10] did not consider the influence of magnetization. Thus, Shi [11] proposed a new plastic-effective magnetic field model that considered the influence of magnetization and plastic deformation. The above models have greatly promoted the development of magneto–mechanical theory. However, the magneto–mechanical model that reflects the relative permeability under elasto–plastic deformation also has some limitations in describing the magnetic signal (e.g., limited application range, the inappropriate initial relative permeability, etc.). Therefore, many efforts should be made to study the magneto–mechanical model that reflects the relative permeability of materials, which can evaluate the relationship between elasto–plastic deformation and magnetic field.

Numerical simulation is an important method to study the detection mechanism of ENDT and to improve the detection efficiency, and the method in which the magneto–mechanical coupling model is organically combined with the numerical simulation method is of great significance to improve the simulation accuracy. At present, numerical simulation methods such as the finite element method (FEM) [1,7,9,12,13], boundary element method (BEM) [14], and magnetic charge method (MCD) [15] are widely used to calculate the distribution of the magnetic field under stress concentration and defects in materials. Especially, FEM simulation is more commonly used to analyze the magneto–mechanical coupling effects of ferromagnetic materials [1,12]. For example, Yuan et al. [1] used a finite element model to simulate the magnetic field distribution around the crack. Yao et al. [12] obtained the residual magnetic field (RMF) signal under the plastic deformation of ferromagnetic material through finite element simulation. In further research, some researchers [9,13,16] have made some efforts to combine magneto–mechanical coupling models with finite element analysis. Shi et al. [9] combined finite element and magneto–mechanical models to establish a quantitative relationship between material defects and magnetic signals. Yao et al. [13] simulated the RMF signals under plastic deformation by setting the relative permeability and coercivity for different regions of the specimen. Sun et al. [16] introduced a stress-induced magnetic constitutive model in the finite element simulation and proposed a method to identify the stresses and defects based on the simulation signal. However, the computational accuracy of the above studies still needs to be improved, and some theoretical models cannot be directly used in analyzing the magnetic signal of the specimen under plastic deformation, which limits the development of quantitative ENDT simulation. In addition, the accuracy of the magneto–mechanical model has a significant influence on the accuracy of the simulation results. Therefore, it is important to study numerical analysis methods that include the magneto–mechanical model and simulation methods to improve the accuracy of simulation results and extend the application scope of numerical simulation.

This paper proposed a magneto–mechanical coupling model that can reflect the dependence of relative permeability on elasto–plastic deformation and a finite element–infinite element coupling method that can replace the traditional finite-element truncation boundary. The proposed magneto–mechanical coupling model and finite element–infinite element coupling analysis method are validated through previous experimental results. The variation of transient magnetic flux signal under elasto–plastic deformation is analyzed, and the quantitative relationship between the peak–peak value signal and elasto–plastic deformation is obtained. This work is useful for improving the accuracy of ENDT.

## 2. Theoretical Analysis

**Figure 1:**
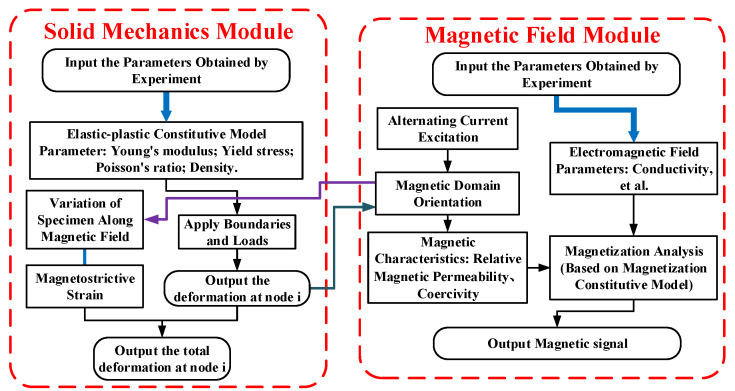
Schematic diagram of magnetic field and solid mechanics coupling simulation.

In the numerical analysis of ENDT, the mechanical finite element, electromagnetic finite element, and theoretical model together constitute the numerical theory of ENDT, and the coupling process of the stress–magnetic analysis is shown in Figure 1. In this paper, the core of the numerical simulation translates the effects of elastic or plastic deformation into changes in the relative permeability and remanence magnetization by means of the magneto–mechanical coupling model and the nonlinear magnetization model. The stress distribution of the specimen can be obtained by the mechanical finite element, and the relative permeability distribution in a material can be obtained based on the magneto–mechanical coupling model, then the remanence magnetization can be obtained based on the nonlinear magnetization model [3], and finally, the magnetic signals of a specimen can be obtained by electromagnetic field analysis. Therefore, the mechanical finite element is first analyzed, then the finite element–infinite element coupling method that can be used for electromagnetic field analysis is introduced, and finally, the magneto–mechanical coupling model that can reflect the relative permeability of materials under elasto–plastic deformation is developed.

### 2.1. Mechanical Analysis

In mechanical finite element analysis, the equilibrium equation of ferromagnetic material under applied load can be written as [9,17]

(1) −∇·σ=F

where σ is the stress tensor, *F* is the applied load vector of the specimen, and the boundary condition can be expressed as [9,17]

(2) $$\left\{\begin{array}{c} \sigma_{s}\cdot n=\bar{F}\\ \mu_{s}=\bar{\mu } \end{array}\right.$$

where n is the normal direction of boundary, $\bar{F}$ is the applied load on the boundary, $\bar{\mu }$ is the applied displacement on the boundary. The distribution of stress in the specimen can be obtained by using the finite element method based on the above equations.

### 2.2. Electromagnetic Field Finite Element–Infinite Element Coupling Analysis

The infinite elements are more suitable for solving infinite region problems than finite element analysis. The essence of an infinite element is an extension of finite elements in an infinite or semi-infinite region problem, and the difference with the finite element is that the truncation boundary of the traditional finite element model, such as the Dirichlet boundary and mixed boundary, is replaced by the infinite element [18,19]. Therefore, in the simulation of ENDT, the specimen and the inner air layer are set as the finite element regions, and the outer air layer is set as the infinite element region.

Maxwell’s equations established by J.C. Maxwell can describe electromagnetic phenomena comprehensively and accurately, which are the basic equations commonly used in electromagnetic field analysis [17,20]. The specific form of Maxwell’s equations is as follows:

(3) $$\left\{\begin{array}{c} \nabla \times H=J_{0}\\ \nabla \cdot B=0 \end{array}\right.$$

where *H* is a magnetic field, *J*_0_ is the current density, *B* is magnetic induction density, B = *μH* (where μ is magnetic permeability, μ = μ_0_μ_r_, μ_0_ is the vacuum magnetic permeability, and μ_r_ is the relative permeability). In the alternating excitation, $J_{0}=\frac{{NV}_{coli}}{A_{coli}R_{Coli}}$ (where *N* is the coil turns number, *V_coli_* is the excitation voltage of coil, *R_coli_* is coil resistance, and *A_coli_* is the cross-sectional area of coil wire). In addition, B=∇×A  (A is the vector potential, ∇·A=0) [21,22], and Equation (3) can be written as $-\frac{1}{\mu }\nabla^{2}$*A*= *J*_0_, which can be calculated by the finite element. The detailed calculated process is shown in Appendix A, and the results is $\frac{1}{\mu }K_{ij}^{1}A_{e}=F_{J0}$, where $F_{J0}=\int_{\Omega }J_{0}\cdot N_{j}d\Omega ,\;K_{ij}^{1}$ is the stiffness matrix, $A_{e}=\left(A_{1},A_{2},\right.\ldots ,A_{i}{)}^{T}$.

On the finite element boundary, the magnetic potential extends to infinity through the infinite element and decays to zero at infinity, thus there is no need to consider the boundary conditions of the magnetic field. The calculation process of the infinite element is shown in Appendix A. In a simulation process, to fit the complex specimen better, the hexahedral finite element of the specimen can be subdivided into tetrahedral finite elements, and the total nodes number of tetrahedral finite elements of the specimen is the same as that of the original hexahedral nodes to facilitate coupling with the infinite element hexahedron [19]. Further, the finite element–infinite element coupling process is only necessary to add the stiffness matrix values calculated by finite elements $K_{ij}^{I}$ and infinite elements $K_{ij}^{2}$, respectively, to the corresponding position of the total stiffness matrix according to node numbers, which realizes the effective coupling of two elements and two methods.

(4) $$\left[\begin{array}{ccc} \cdots & \cdots & \cdots \\ \cdots & K_{ij} & \cdots \\ \cdots & \cdots & \cdots \end{array}\right]=\left[\begin{array}{ccc} \cdots & \cdots & \cdots \\ \cdots & K_{ij}^{1} & \cdots \\ \cdots & \cdots & \cdots \end{array}\right]=\left[\begin{array}{ccc} \cdots & \cdots & \cdots \\ \cdots & K_{ij}^{2} & \cdots \\ \cdots & \cdots & \cdots \end{array}\right]$$

Infinite elements are used at the boundary of finite elements, which avoids the distortion of calculation results caused by the truncation boundary of finite elements. In numerical simulations, an open-source solver Pardiso with good performance and high parallelization is used, which can be directly solved by LU decomposition.

### 2.3. Magneto–Mechanical Coupling Theory

Ferromagnetic materials exhibit a complex nonlinear relationship between stress and the magnetic field during the magnetization process. Many physical models have been established to describe the variation of magnetostrictive strain, magnetization, and hysteresis of ferromagnetic materials with stress and magnetic field [2,3,4,5,6,9,23]. Among them, the Z-L model [5] with significant physical significance and the Jiles–Atherton model (J-A model) [23] with a concise form are more widely used. Relatively speaking, the model in the literature [3,4] has higher accuracy in quantitatively describing the magneto-elastoplastic coupling behavior of materials. In addition, Shi [6] proposed a new concise, accurate, and easy-to-calculate magneto-elastic model, which greatly improved the computational and application capabilities of the model. Therefore, based on the research [3,4,5,6], the magneto–mechanical effect can be further researched (Appendix B and Appendix B.1).

The effective magnetic field under the combined action of stress and the magnetic field can be divided into two parts. The first part is caused by the movement of the domain wall

$$\frac{2M}{\mu_{0}M_{ws}^{2}}\left[\lambda_{s}\sigma -\Lambda (\sigma )\right]\;(where\;\Lambda_{0}(\sigma )=\frac{\lambda_{s}}{4}\;\left[\sigma +\frac{{3\sigma }_{s}}{\beta }In\;cosh\left(\frac{\beta \sigma }{\sigma_{s}}-archan\;h\frac{1}{3}\right)\right],\;M)$$

where, *M* is magnetization, *λ_s_* is saturation magnetostrictive, *M_ws_* is saturation magnetization contributed by the movement of the magnetic domain wall under an unstressed state, $\sigma_{s}$ is the yield stress, and *β* is a scale factor associated with the growth rate of the nonlinear elastic strain) [24,25], and the other part is caused by the rotation of magnetic domain wall $-\frac{2\vartheta \lambda_{s}\sigma k}{\mu_{0}}\;\frac{\left(1-\tau \right)M}{\left(M_{s}-M_{0}{)}^{2}\right.}$ where ϑ is the jump factor related to the change in magnetic domain structure, *k* is a scaling factor related to the rotation of the magnetic domain wall, τ=M0Ms,M0  is the stress-dependent saturation wall-displacement magnetization, $M_{0}=\frac{1}{3}M_{ws}\left[1-tanh\left(\beta \frac{\sigma }{\sigma_{s}}-\arctan h\left(\frac{1}{3}\right)\right)\right]$, and *M_s_* is the saturation magnetization) [24,25,26]. The initialization parameters (such as initial magnetic susceptibility $\chi_{in}$, demagnetization factor, etc.) dependent on materials are normalized as ξ=δχin where *δ* is normalization coefficient obtained by fitting the material initialization parameters) [7]. Therefore, the relationship between the effective magnetic field *H_e_*, magnetization *M*, and elastic stress can be written as

(5) $$H_{e}=\xi M+\frac{2M}{\mu_{0}M_{ws}^{2}}\left[\lambda_{s}\sigma -\Lambda (\sigma )\right]-\frac{2\vartheta \lambda_{s}\sigma k}{\mu_{0}}\;\frac{\left(1-\tau \right)M}{\left(M_{s}-M_{0}{)}^{2}\right.}$$

According to the three-dimensional model construction method of isotropic materials in the literature [2], the above one-dimensional nonlinear effective field equation can be extended to three-dimensional space:

(6) $$\left\{\begin{array}{c} H_{ex}\\ H_{ey}\\ H_{ex} \end{array}\right\}=\left[\begin{array}{ccc} \xi_{xx} & 0 & 0\\ 0 & \xi_{yy} & 0\\ 0 & 0 & \xi_{zz} \end{array}\right]+\left\{\begin{array}{c} M_{x}\\ M_{y}\\ M_{z} \end{array}\right\}+\frac{2}{\mu_{0}M_{ws}^{2}}\left[\begin{array}{ccc} \lambda_{s}{\hat{\sigma }}_{xx\;}{-\Lambda }_{0}\left({\hat{\sigma }}_{xx}\right) & {\hat{\sigma }}_{xx} & {\hat{\sigma }}_{xz}\\ {\hat{\sigma }}_{xy} & \lambda_{s}{\hat{\sigma }}_{yy}-\Lambda_{0}\left({\hat{\sigma }}_{yy}\right)\; & {\hat{\sigma }}_{yz}\\ {\hat{\sigma }}_{xz} & {\hat{\sigma }}_{yz} & \lambda_{s}{\hat{\sigma }}_{zz\;}-\Lambda_{0}\left({\hat{\sigma }}_{zz}\right) \end{array}\right]\left\{\begin{array}{c} M_{x}\\ M_{y}\\ M_{z} \end{array}\right\}-\frac{2\vartheta \lambda_{s}k\left(1-\tau \right)}{\mu_{0}\left(M_{s}-M_{0}{)}^{2}\right.}\;\left[\begin{array}{ccc} {\hat{\sigma }}_{xx} & {\hat{\sigma }}_{xy} & {\hat{\sigma }}_{xz}\\ {\hat{\sigma }}_{xy} & {\hat{\sigma }}_{yy} & {\hat{\sigma }}_{yz}\\ {\hat{\sigma }}_{xz} & {\hat{\sigma }}_{yz} & {\hat{\sigma }}_{zz} \end{array}\right]\left\{\begin{array}{c} M_{x}\\ M_{y}\\ M_{z} \end{array}\right\}$$

where ${\hat{\sigma }}_{xy},{\;\hat{\sigma }}_{xz},\;{\hat{\sigma }}_{yz}$ is the engineering shear stress in the principal stress space

$${\hat{\sigma }}_{xy}={\hat{\sigma }}_{xz}={\hat{\sigma }}_{yz}=0,\;\Lambda_{0}\left(\sigma_{mm}\right)=\frac{\lambda_{ws}}{4}\left[\sigma_{mm}+\frac{{3\sigma }_{s}}{\beta }\;In\;cosh\;\left(\frac{{\beta \sigma }_{mm}}{\sigma_{s}}-arctan\;h\frac{1}{3}\right)\right]\cdot$$

It can be seen from Equation (6) that the magnetic field and magnetization in different directions are uncoupled with each other. Combining the relationship between the magnetic field and susceptibility, the susceptibility in different directions can be obtained:

(7) $$\left\{\begin{array}{c} \frac{1}{x}=\xi_{mxx}+\frac{2}{\mu_{0}M_{ws}^{2}}\left[\lambda_{s}\sigma_{xx}-\Lambda_{0}\left(\sigma_{xx}\right)\right]-\frac{{2\theta \lambda }_{s}k\left(1-\tau \right)\sigma_{xx}}{\mu_{0}\left(M_{s}-M_{0}{)}^{2}\right.}\\ \frac{1}{x_{y}}=\xi_{my}+\frac{2}{\mu_{0}M_{ws}^{2}}\left[\lambda_{s}\sigma_{yy}-\Lambda_{0}\left(\sigma_{yy}\right)\right]-\frac{{2\theta \lambda }_{s}k\left(1-\tau \right)\sigma_{yy}}{\mu_{0}\left(M_{s}-M_{0}{)}^{2}\right.}\\ \frac{1}{x_{z}}=\xi_{mzz}+\frac{2}{\mu_{0}M_{ws}^{2}}\left[\lambda_{s}\sigma_{zz}-\Lambda_{0}\left(\sigma_{zz}\right)\right]-\frac{{2\theta \lambda }_{s}k\left(1-\tau \right)\sigma_{zz}}{\mu_{0}\left(M_{s}\right.-M_{0}{)}^{2}} \end{array}\right.$$

In theoretical calculations, the relative permeability of the material can be obtained based on the relational expression $\mu_{r}=1+\chi$.

(8) $$\left\{\begin{array}{c} \mu_{rx}=1+1/\left\{\xi_{mxx}+\frac{2}{\mu_{0}M_{ws}^{2}}\left[\lambda_{s}\sigma_{xx}-\Lambda_{0}\left(\sigma_{xx}\right)\right]-\frac{{2\theta \lambda }_{2}k\left(1-\tau \right)\sigma_{xx}}{\mu_{0}\left(M_{s}-M_{0}{)}^{2}\right.}\right\}\\ \mu_{ry}=1+1/\left\{\xi_{myy}+\frac{2}{\mu_{0}M_{ws}^{2}}\left[\lambda_{s}\sigma_{yy}-\Lambda_{0}\left(\sigma_{yy}\right)\right]-\frac{{2\theta \lambda }_{s}k\left(1-\tau \right)\sigma_{yy}}{\mu_{0}\left(M_{s}-M_{0}{)}^{2}\right.}\right\}\\ \mu_{rz}=1+1/\left\{\xi_{mzz}+\frac{2}{\mu_{0}M_{ws}^{2}}\left[\lambda_{zz}\sigma_{zz}-\Lambda_{0}\left(\sigma_{zz}\right)\right]-\frac{{2\theta \lambda }_{2}k\left(1-\tau \right)\sigma_{zz}}{\mu_{0}\left(M_{s}-M_{0}{)}^{2}\right.}\right\} \end{array}\right.$$

#### Magneto–Plastic Coupling Model

The previous theoretical models [24,27] describe the effects of plastic deformation on magnetic signals by introducing a shape coefficient, pinning coefficient, and molecular field coefficient, which also introduces many unknown and difficult-to-obtain parameters that make it difficult to be directly applied in engineering analysis. Compared with previous models [24,27], an explicit equation between the effective magnetic field and plastic deformation is described in the literature [3,11], which is more accurate and has fewer parameters. The relationship between the plastic deformation and effective magnetic field can be written as

(9) $$H_{\varepsilon }^{p}=-{\kappa \varepsilon }_{p}^{n}(H+M)$$

where $\varepsilon_{p}$ is the plastic deformation, κ is a constant related to the material properties, and n is a fitting coefficient, which represents domain wall pinning that varies with the evolution of dislocation morphology under plastic deformation [11].

The applied magnetic field *H* during the magnetization process is much less than the magnetization *M*, thus it can be ignored in simulation analysis, and the Equation (9) can be expressed as

(10) $$H_{\varepsilon }^{p}=-{\kappa \varepsilon }_{p}^{n}M$$

Considering the molecular coupling field α and demagnetizing field *N_d_* in the plastic stage, the relationship between plastic deformation and magnetization can be written as

(11) $$H_{\varepsilon -t}^{p}=\left(\alpha -N_{d}\right)M-{\kappa \varepsilon }_{p}^{n}M$$

Ferromagnetic materials enter the plastic stage from the elastic stage, the dislocations and lattice slip inside the material increase, the number of pinning points increases dramatically, and the magnetic domains undergo irreversible reorientation, resulting in a sharp change in relative permeability, which is also supported by the experimental results of Iordache et al. [28]. Ma et al. [29] introduced a model with maximum relative permeability in the elastic stage as a revised factor to describe the variation of relative permeability with plastic deformation, which can analyze the magneto–mechanical coupling problem under small plastic deformation. However, the model in the literature [29] is based on a bilinear isotropic hardening model, which describes the linear variation of relative permeability with large plastic deformation that deviates from experimental results. Therefore, based on the nonlinear model in Equation (11), a revised factor for the change in magnetic properties caused by elastic deformation can be introduced to describe the relative permeability under plastic deformation [29].

(12) $$\mu_{r\varepsilon }=\overset{´}{\mu }-\frac{1}{\left(\alpha -N_{d}\right)}-{\kappa \varepsilon }_{p}^{n}$$

where $\mu^{\prime }=\omega \mu_{max},\;\;\omega$ is the material-dependent fit coefficient and $\mu_{max}$ is the maximum relative permeability.

It is worth noting that the effect of elastic or plastic deformation on the magnetic property of ferromagnetic materials is expressed as the variation of magnetic parameters (e.g., magnetic susceptibility, relative permeability, and remanence magnetization) with stress, and the parameters of the theoretical model can be obtained by fitting the experimental results of the specimen without a defect under different loads. The relationship between the stress and the remanence magnetization after loading can be obtained based on the model in [3], which is combined with the model proposed in this paper to calculate the magnetic fields on the surface of the specimens. What calls for special attention is that Equations (8) and (12) are a 3D model and 1D model based on a 3D elastic effective magnetic field equation and a 1D plastic effective magnetic field equation, respectively, and their application scope is different.

## 3. Validation of the Numerical Method and the Proposed Model

### 3.1. Finite Element–Infinite Element Coupling

**Figure 2:**
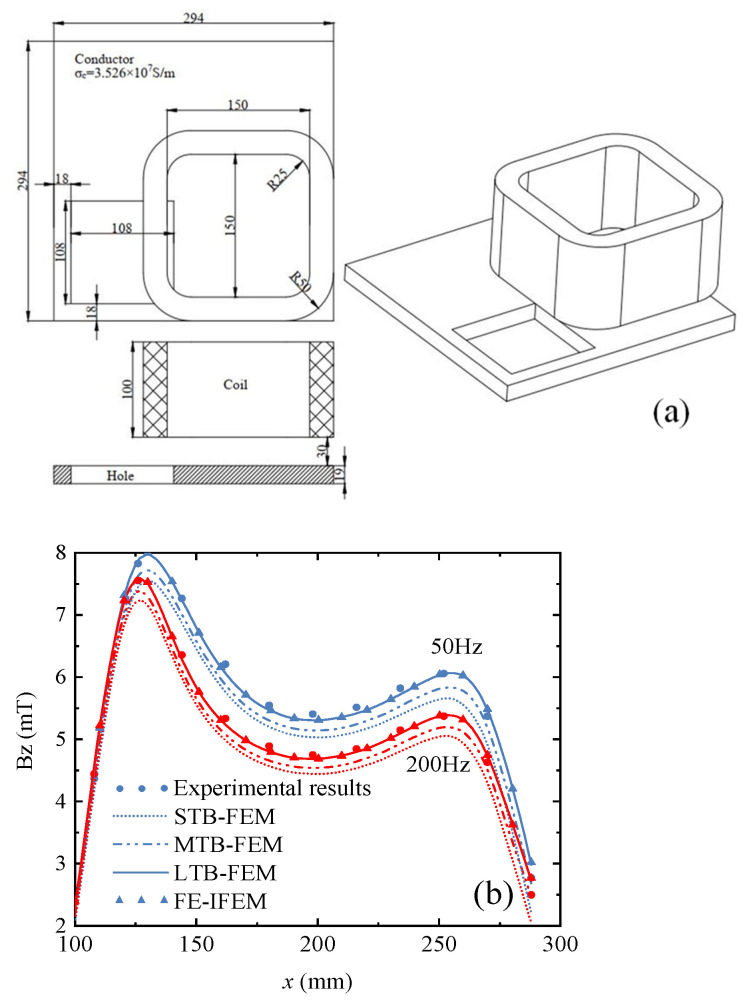
The computation model of TEAM Problem 7 and the comparison results. (**a**) Computation model of TEAM Problem 7, (**b**) Magnetic flux density *B_z_*.

To verify and evaluate the advantages and disadvantages of various numerical algorithms, the international Testing Electromagnetic Analysis Methods (TEAM) workshop has proposed many benchmark problems for electromagnetic field analysis, and the theoretical solutions and experimental data are recognized as authoritative criteria for the evaluation of new methods. Among them, the TEAM Problem 7 benchmark problem is a transient electromagnetic computational model consisting of a rectangular current-carrying coil placed in air and a perforated aluminum plate under the coil, which is energized with a sinusoidal current to simulate the leakage magnetic field in the vicinity of components, and then the experimental values of the leakage magnetic field along the line (*y* = 72 mm, *z* = 34 mm) were measured and compared with the simulation results to verify the accuracy of the numerical analysis method. Therefore, the finite element–infinite element coupling method in this paper is also validated using TEAM Workshop Problems 7 [30]. The detailed information on the numerical model for TEAM Problem 7 is shown in Figure 2a. The excitation current of the coil is 2742 Ampere, and the frequencies of the current are 50 Hz and 200 Hz.

Figure 2b shows a comparison of the experimental values measured along the line (*y* = 72, *z* = 34 mm) with the simulation results of different simulation methods. The simulation method adopts the conventional artificial boundary finite element methods (e.g., the large truncation boundary–finite element method (LTB-FEM), middle truncation boundary–finite element method (MTB-FEM), small truncation boundary–finite element method (STB-FEM)), and finite element–infinite element method (FE-IFEM) proposed in this paper. It can be seen from Figure 2b that the simulation results are consistent with the experimental results [30], but there are differences in the accuracy of different simulation methods.

To quantitatively analyze the errors of different methods, the normalized mean square error (NMSE) $NMSE=\sum_{1}^{n}\left(y_{i}-{\hat{y}}_{i}{)}^{2}/\sum_{1}^{n}{\hat{y}}_{i}^{2}\right.$ (where $y_{i}$ is the simulation results and ${\hat{y}}_{i}$ is the experimental results) is introduced in this paper. As shown in Table 1, it is obvious that the NMSE of FE-IFEM is lower than that of LTB-FEM, MTB-FEM, and STB-FEM. The NMSE of the LTB-FEM is closest to the FE-IFEM, but the computation time of the LTB-FEM is 3.86 times that of the FE-IFEM. Therefore, the FE-IFEM can improve the efficiency of electromagnetic field simulation analysis.

### 3.2. Magneto–Elastic Coupling Model

To verify the reliability of the proposed model, the inverse of the relative permeability calculated by the different theoretical models is compared with the experimental data [28], and the comparison theoretical models adopted are Sun’s model [7] and Yang’s model [31]. The parameters of the model proposed in this paper are set as *ξ_mxx_* = 0.00545, *λ_s_* = 4.6 × 10^−6^, *M_ws_* = 0.45 × 10^6^ A/m, *M_s_* = 0.608 × 10^6^ A/m, *σ_s_* = 150 MPa, *k* = 1, *ϑ* = 0.45, *β* = 1.

**Figure 3:**
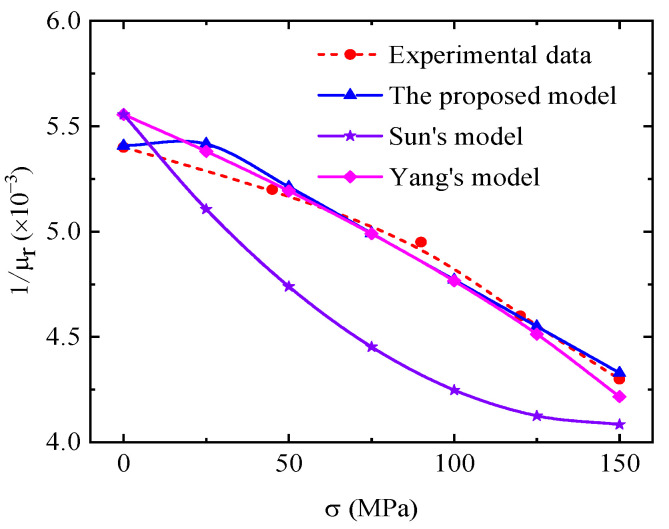
Comparison between the inverse of the relative permeability calculated by different models and the experimental results [28].

It can be seen from Figure 3 that the inverse of the relative permeability decreases nonlinearly with the increase in elastic stress. Compared with the experimental results [28], the above three models can reflect the nonlinear variation of the relative permeability with stress. The NMSEs of the proposed model, Yang’s model [31], and Sun’s model [7] are 0.015%, 0.026%, and 0.66%, respectively. Therefore, compared with Sun’s model [7] and Yang’s model [31], the proposed model has a higher consistency between the prediction results and the experimental results [28].

To test the application scope of the model proposed in this paper, the experimental data between magnetic signal and elasto–plastic deformation of ferromagnetic materials are compared with theoretical results calculated by the FE-IFEM. In the elastic deformation section, the magnetic signals of different material specimens, such as X80 steel and Q345 steel, are simulated using the FE-IFEM. Hereby, the experimental results [32] on the relationship between the magnetic signal and elastic stress for Q345 steel are used to verify the reliability of the proposed model, and the geometry and dimension of the Q345 steel specimen used for simulation are in accordance with the literature [32]. The parameters of the proposed model are set as *ξ_mxx_* = 0.0028, *λ_s_* = 4.6 × 10^−6^, *M_ws_* = 1.2 × 10^6^ A/m, *M_s_* = 1.512 × 10^6^ A/m, *σ_s_* = 400 MPa, *k* = 2.8, *ϑ* = 0.75, *β* = 1.

**Figure 4:**
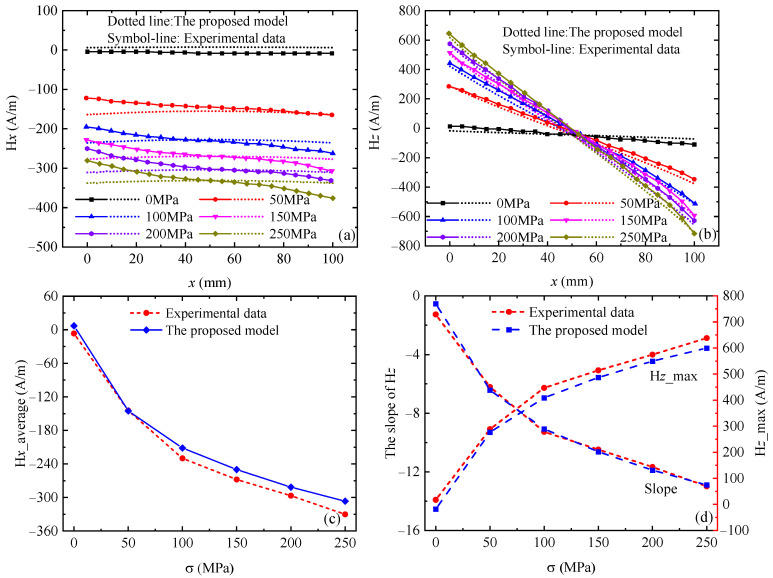
Comparison between the theoretical calculation results of the proposed model and the experimental data [32]. (**a**) Tangential component H*x*, (**b**) normal component H*z*, (**c**) the average of H*x*, (**d**) the slope and maximum of H*z*.

Figure 4 shows the comparison between the calculation results of the proposed model and the experimental data [32]. The experimental results [32] show that the tangential component H*x* is an approximately constant function, and the curves of H*x* move downward with the increase in stress. The absolute value of H*x* increases, but the moving rate decreases, which shows a nonlinear increase in the magnetic field value with respect to the stress. The normal component H*z* exhibits a good linear relationship with respect to the specimen axis, the curve turns clockwise, the absolute value of the slope increases, the rotation amplitude of the magnetic field curve becomes smaller, and the distribution is more concentrated. As shown in Figure 4, the theoretical calculation results of the proposed model are in good agreement with the experimental results [32]. As shown in Figure 4c,d, the absolute value of H*x*_average (the average of H*x*), H*z*_slope (the slope of H*z*), and H*z*_max (the maximum value of H*z*) increases nonlinearly with the increase in stress, and the simulation results are coincident with the experimental results [32]. The phenomenon shown in Figure 4 is due to the fact that the elastic stress promotes the unpinning of the domain wall, which rotates the magnetic moment in the direction of easy magnetization, thereby increasing the overall magnetic field. As shown in Figure 4, there is a certain difference between the experimental results [32] and the theoretical calculation results. This may be because the experimental results [32] are affected by ferromagnetic fixtures, incomplete demagnetization, and measurement errors, while the theoretical calculation results are obtained under ideal conditions without considering any interference factors. However, the theoretical calculation results have the same characteristics and trends as the experimental results [32], which proves the validity of the theoretical model proposed in this paper.

To quantify the accuracy of the calculation results of each model, the NMSE between the theoretical calculation results of different models (the proposed model, Sun’s model [7], and Yang’s model [31]) and the experimental results [7] is calculated in this paper, and the results of the NMSE calculation for each model are shown in Table 2.

From Table 2, it can be seen that the NMSE average of the tangential component H*x* of the proposed model, Sun’s model [7], and Yang’s model [31] is 7.96%, 14.07%, and 9.61%, respectively. The NMSE average of the normal component H*z* of the proposed model, Sun’s model [7], and Yang’s model [31] is 40.26%, 49.16%, and 48.15%, respectively. Overall, compared to Sun’s model [7] and Yang’s model [31], the theoretical results of the proposed model are closer to the experimental results [7]. Compared with Sun’s model [7] and Yang’s model [31], the proposed model is derived from the magneto–mechanical model, and the physical basis is the motion theory of magnetic domains of ferromagnetic materials under stress, which also considers the change in magnetization of the rotation of magnetic domain wall. Therefore, the proposed model in this paper can effectively describe the magneto–mechanical coupling effect of ferromagnetic materials, i.e., the proposed model can more accurately describe the nonlinear magnetization behavior under the excitation of magnetic fields.

### 3.3. Magneto–Plastic Coupling Model

In the plastic state, the experimental results [33] of Q345R are used to verify the validity of the magneto–plastic model, and the geometry and dimension of the specimen used for the simulation are consistent with the literature [33]. The parameters of the proposed model are set as *α* = 1 × 10^−4^, *N_d_* = 2.5 × 10^−3^, *κ* = 2 × 10^−34^, *μ’* = 1365, *n* = 4, and *σ_s_* = 345 MPa.

**Figure 5:**
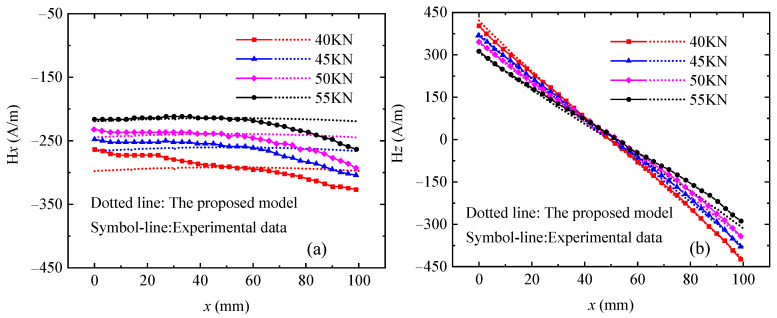
Comparison between the theoretical results and the experimental data [33]. (**a**) The tangential component H*x*, (**b**) the normal component H*z*.

Figure 5 shows a comparison between the theoretical calculation results of the proposed model and the experimental data [33]. It can be seen that the tangential component H*x* moves upward with the increase in the tensile loading, and the normal component H*z* curve turns counterclockwise around the center point of the specimen, which is opposite to the elastic stage. It is worth noting that the changes in H*x* and H*z* are nonlinearly related to loads. Meanwhile, in Figure 5b, the zero-crossing point of H*z* is concentrated into one position during plastic deformation, while there is an irregular drift of the zero-crossing point under tensile loads after elastic deformation.

The interatomic distance is changed by an applied stress, leading to a reorientation of magnetic domains along with tensile stress or perpendicular to compressive stress direction, thus causing a change in the magnetic behavior of ferromagnetic materials, which is known as a piezomagnetic effect [34]. Based on the piezomagnetic effect, when a magnetic field is along the applied stress axis, the specimen forms a magnet similar to a magnet with opposite poles at both ends. The variation of normal component H*z* with stress in the plastic stage is different from that in the elastic stage, which is because the specimen still has a certain tensile elongation after removing the applied stress, resulting in the residual compressive stress in the specimen. The magnetization moment orientation is changed to a direction perpendicular to the residual compressive stress. Consequently, the stress-induced effective magnetic field is reduced, as shown in Figure 5b. In conclusion, the proposed model can effectively describe the complex magnetic behavior of materials under plastic deformation.

## 4. The Effect of Elasto–Plastic Deformation on Transient Magnetic Flux Signal

### 4.1. Simulation of Transient Magnetic Flux Signal Under Elastic Deformation

**Figure 6:**
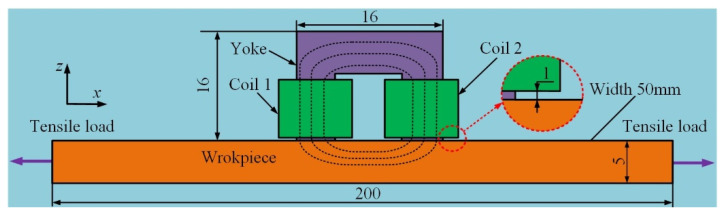
The geometry and dimension of the simulation model.

In the following section, the proposed model is applied to perform a theoretical analysis of magnetic flux signals based on the FE-IFEM. The parameters of the proposed model are consistent with those of the Q345 steel in Section 3.2. The geometry and dimension of the simulation model are in accordance with the literature [32]. See Figure 6.

The specimen is magnetized by an electromagnet consisting of a U-shaped yoke and two circular driving coils with a dimension of 10 mm × 5 mm (height×radial thickness) wounded on two yoke legs. A sinusoidal AC drive voltage with a frequency of 10 Hz and voltage of 14.5 V is applied to coil 1 to generate an alternative excitation magnetic field. The magnetic field generated by coil 1 is strong enough to drive the yoke material to saturation. The yoke material follows Jiles–Atherton magnetic hysteresis model (J-A model) [35], and the parameters are all diagonal matrices, as shown in Table 3.

**Figure 7:**
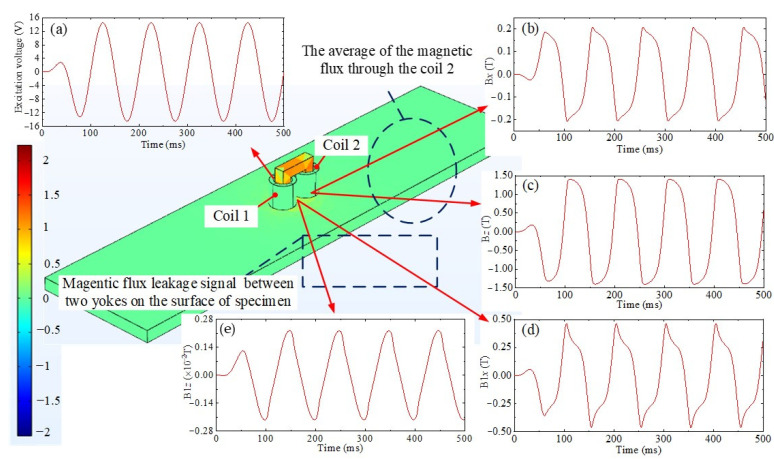
Signals obtained from simulation results. (**a**) Excitation voltage, (**b**) tangential component B*x*, (**c**) normal component B*z*, (**d**) tangential component B1*x*, (**e**) normal component B1*z*.

Figure 7 shows the magnetic flux signal obtained from the simulation process. The AC excitation voltage shown in Figure 7a is applied to coil 1, and the tangential component B*x* and normal component B*z* are the magnetic flux density components through coil 2, as shown in Figure 7b,c, respectively. The tangential component B1*x* and normal component B1*z* are the magnetic flux leakage components at the middle point between the two yokes on the specimen surface, as shown in Figure 7d,e, respectively.

**Figure 8:**
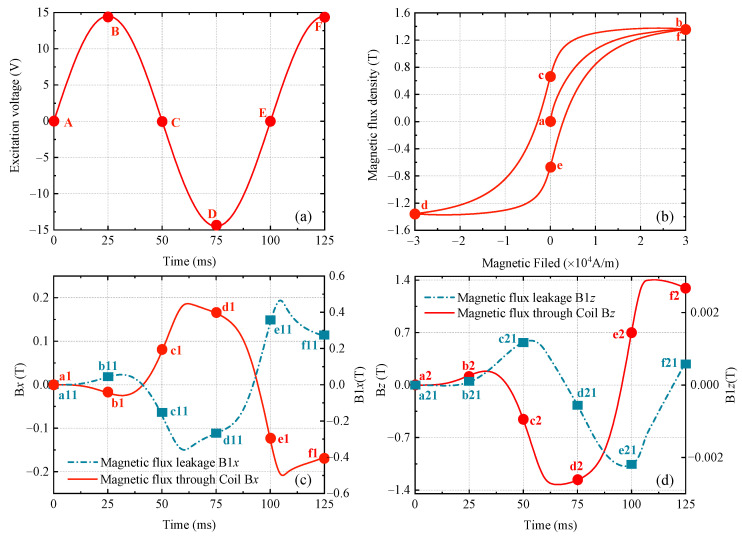
B-H curve and nonlinear magnetic signal variation based on the excitation voltage signal. (**a**) Excitation voltage signal, (**b**) B-H curve, (**c**) tangential component, (**d**) normal component.

To clearly distinguish the feedback signal of the specimen under the excitation voltage at different stages, the analysis must be carried out in combination with the magnetization state of the yoke, and the relationship between the different signals is shown in Figure 8. According to the characteristics of the excitation voltage (the voltage curve in Figure 8a) and the J-A model of the yoke (the hysteresis loop in Figure 8b), the simulation process of ENDT can be divided into different ranges, as shown in Figure 8. The corresponding relationship between the driving voltage, hysteresis loop, and the magnetic signal in different ranges is shown in Table 4. In practice, the range of A-B of the driving voltages in Figure 8a corresponds to the range of A-B in Figure 8b. This stage is the initial magnetization of the yoke, and the magnetic flux signal in this stage is distorted, as shown in the range of a1–b1, a11–b11, a2–b2, a21–b21 in Figure 8c,d. It is worth noting that when analyzing the magnetic signal, the distortion stage of the signal should be avoided, and the signal stabilization stage after the voltage D-E stage should be selected. Therefore, the analysis time range of the feedback signal in this paper is 0–200 ms, and the time for analyzing the peak–peak value time is after 100 ms.

**Figure 9:**
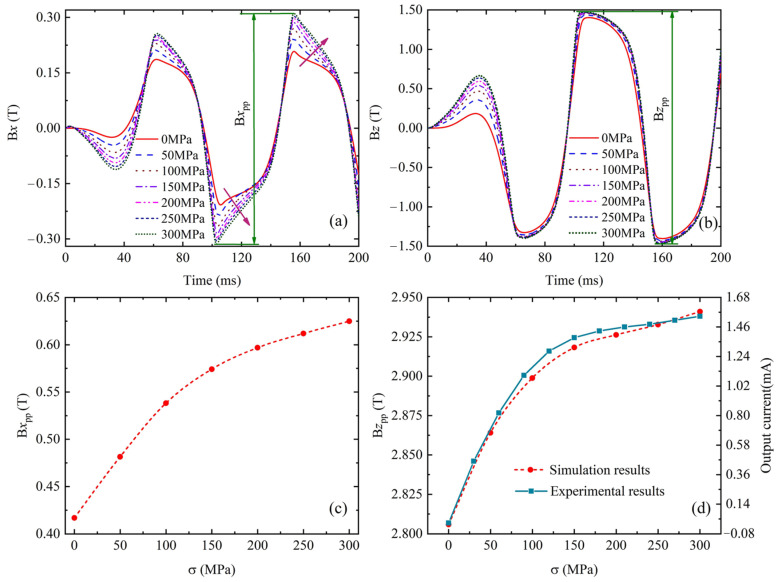
Effects of elastic stress on magnetic flux density through the coil 2. (**a**) Tangential component B*x*, (**b**) normal component B*z*, (**c**) the relationship between B*x*_pp_ and elastic stress, (**d**) the comparison of the relationship between B*z*_pp_ or experimental results and elastic stress.

Figure 9 shows the magnetic flux density through coil 2 under elastic stress. It can be seen from Figure 9 that B*x* and B*z* of the magnetic flux density through coil 2 increase with the increase in elastic stress, and their peak values also increase. To describe the quantitative effect of elastic stress on magnetic flux density through coil 2, some parameters are defined as shown in Figure 9a,b, which are the peak–peak values B*x*_pp_ and B*z*_pp_ for magnetic flux density. The effect of elastic stress on B*x*_pp_ and B*z*_pp_ is shown in Figure 9c,d, and the simulation result shows that B*x*_pp_ and B*z*_pp_ increase nonlinearly with the increase in elastic stress. In the initial stage of elastic deformation, B*x*_pp_ and B*z*_pp_ increased sharply with the increase in elastic stress, and with the stress further enlarged, B*x*_pp_ and B*z*_pp_ increased slowly.

According to the relationship between total magnetic flux Φ and induced current $I_{in}:I_{in}=\frac{N_{in\;}}{R_{t}}\frac{d\Phi }{dt}$ (where $N_{in}$ is the number of the induced coil turns and $R_{t}$ is the resistance), the variation of magnetic flux entering the yoke vertically is proportional to the induced current. Therefore, the output current or voltage measured by the magnetic nondestructive testing equipment can be used to evaluate the reliability of the simulation results, and the comparison between simulation results and experimental results for Q345 steel under elastic stress is also shown in Figure 9d. The comparison results show that the simulation results are in good agreement with the experimental results. In particular, the relationship between *Bz_pp_* and elastic stress is approximately a hyperbolic tangent function. Therefore, a quantitative model of the hyperbolic function relationship between B*z*_pp_ and applied stress is established:

$${Bz}_{pp}={Bz}_{0}+atanh\left(\frac{\gamma \sigma }{\sigma_{s}}\right)$$

where *Bz*_0_ is the initial value of B*z*_pp_ in unstressed states, *a* is a fitting coefficient related to the signal type (e.g., magnetic flux density, magnetic flux leakage), *γ* is a fitting coefficient related to materials, and *σ_s_* is the yield stress. Here, *Bz*_0_ = 2.81, *a* = 0.138, *γ* = 3, and the NMSE between the theoretical calculation results of the quantitative model and the simulation results (Figure 9d) is 3.0093 × 10^−6^.

**Figure 10:**
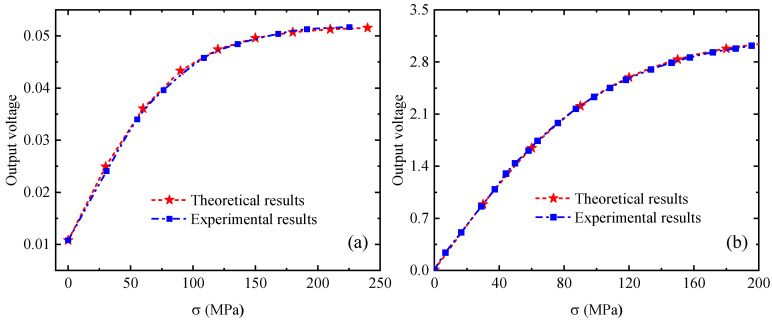
Comparison between the theoretical results and the experimental results [36,37]. (**a**) Langman’s experimental results [36], (**b**) Kenji’s experimental results [37].

To verify the accuracy of the above quantitative model, the theoretical calculation results are compared with the existing experimental results [36,37], as shown in Figure 10. It can be seen from Figure 10 that the theoretical calculation results are in good agreement with the experimental results, which proves the validity and correctness of the quantitative model proposed in this paper.

The effect of elastic stress on the magnetic flux leakage at the middle point between the two yokes on the specimen surface is consistent with the variation law shown in Figure 9, and they also coincide with the research results [7,31]; therefore, it is not repeated here. The reason for the above phenomenon shown in Figure 9 and Figure 10 is that elastic stress promotes the unpinning of the domain walls and rotates the magnetic moment in the easy magnetization direction, thereby increasing the magnetic flux signal. Meanwhile, the quantitative model established in this paper can accurately describe the variation of the normal magnetic signals with elastic deformations.

### 4.2. Simulation of Transient Magnetic Signals Under Plastic Deformation

The parameters of the proposed model are consistent with those of Q345R steel (*σ_s_* = 345 MPa) in Section 3.3, and the geometry and dimension of the simulation model are in accordance with the literature [32].

**Figure 11:**
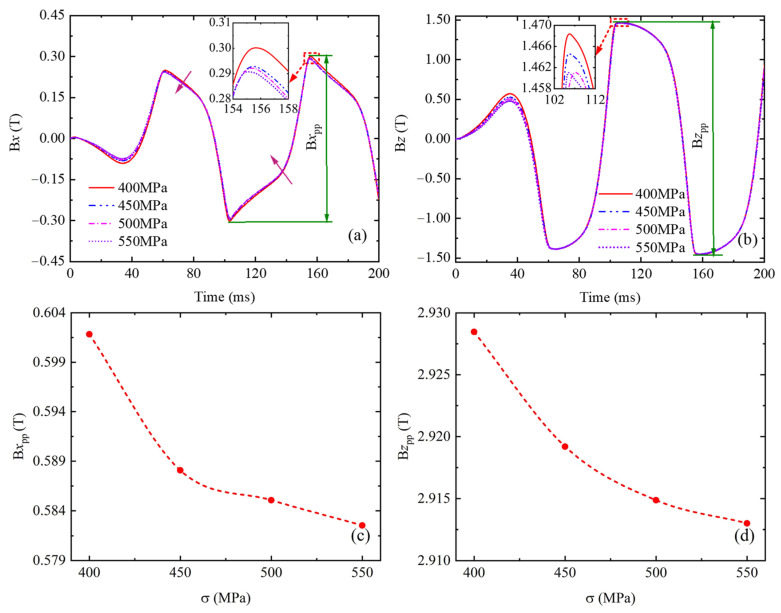
Effects of plastic stress on magnetic flux density through coil 2. (**a**) Tangential component B*x*, (**b**) normal component B*z*, (**c**) the relationship between B*x*_pp_ and plastic stress, (**d**) the relationship between B*z*_pp_ and plastic stress.

Figure 11 shows the effect of plastic deformation on the magnetic flux density of coil 2. As shown in Figure 11, B*x* and B*z* decrease with the increase in plastic deformation, and their peak value also decreases with the increase in plastic deformation. B*x*_pp_ and B*z*_pp_ decrease more dramatically in the plastic deformation range from 400 MPa to 450 MPa, but the downtrend becomes smooth for larger plastic deformation. The variation law of B1*x*, B1*z*, B1*x*_pp_, and B1*z*_pp_ with plastic deformation is consistent with Figure 11.

The reasons for the phenomena shown in Figure 11 are very complicated. For now, a generally accepted theory is the interaction between dislocations and domain walls [38]. With the increase in plastic stress, the dislocation density increases, and dislocation distributes heterogeneously, resulting in different dislocation configurations. The isolated dislocation is uniformly distributed in the grain boundary region rather than in the grain interior during the early deformation stage. With the increase in plastic stress, the dislocation density gradually increases and forms the dislocation tangles. In further deformation, the dislocation tangles are interconnected and form a cellular structure. Therefore, the plastic deformation exhibits a different mechanism of domain walls interacting with isolated dislocations, dislocation tangles, or dislocation cellular structure, which depends on the degree of plastic deformation. During initial plastic deformation, uniformly distributed isolated dislocations and small dislocation tangles hinder the domain wall ordering motion, thereby forming domain wall pinning points. As the plastic deformation increases, dislocation tangles increase, and dislocation cellular structures are gradually generated, which can form very strong pinning sites that cannot be unpinned by the applied stress, and a large number of magnetic domain walls become immovable, resulting in a decrease in the irreversible magnetization and, consequently, the reduction in the specimen magnetic field with the plastic deformations [39].

According to the characteristics of magnetic signal amplitude under plastic deformation and the previous research [39,40], a quantitative model of the exponential function relationship between B*z*_pp_ and plastic deformation is established:

$${Bz}_{pp}^{p}={Bz}_{0}+a_{p}e^{\frac{\gamma_{p^{\sigma }}}{\sigma_{s}}}$$

where *Bz*_0_ is the initial value of B*z*_pp_ in plastic deformation, *a_p_* is a fitting coefficient related to the signal type (e.g., magnetic flux density, magnetic flux leakage), and *γ_p_* is a fitting coefficient related to materials. Here, *Bz*_0_ = 2.911, *a_p_* = 6, and *γ_p_* = −5.03.

**Figure 12:**
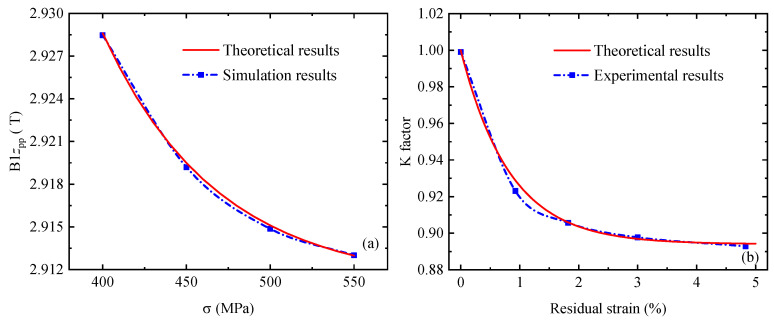
Comparison between the quantitative results and simulation or experimental results [40]. (**a**) Experimental result of *B*1*z*_pp_ [40], (**b**) Experimental result of K factor [40].

The comparison between the simulation results (Figure 11d) and the quantitative results is shown in Figure 12a, and the NMSE between the theoretical calculation results of the quantitative model and the simulation results is less than 0.001%. In addition, the theoretical calculation results are compared with the existing experimental results [40], as shown in Figure 12b. It can be seen from Figure 12 that the theoretical calculation results are in good agreement with the experimental results, which proves the validity and correctness of the quantitative model proposed in this paper.

## 5. Conclusions

This paper proposed a new magneto–mechanical model and a finite element–infinite element coupling method. The parameters of the proposed model can be determined by the experimental data. Several conclusions can be obtained as follows.

(a) An explicit magneto-elastoplastic coupling model that reflects the dependence of relative permeability on elasto–plastic deformation is obtained, and its simulation results are closer to the experimental results than the existing models. The validities of the proposed finite element–infinite element coupling method are verified by the experimental result of TEAM Problem 7.

(b) Compared with the existing model, the NMSE between the theoretical calculation results of the model proposed in this paper is the smallest, which indicates that the proposed model is in good agreement with the previous experimental results and proves that the model proposed in this paper has a good predictive ability. It is worth noting that the model proposed in this paper is derived from the magneto–mechanical model, in which the magnetization and magnetic field are nonlinear functions with respect to the stress, and the combination of the remanence magnetization can more accurately describe the nonlinear magnetization behavior under the excitation of constant and alternating magnetic fields.

(c) The transient magnetic flux signal of a specimen under elasto–plastic deformation is simulated. The simulation results show that the magnetic flux density increases nonlinearly with the increase in elastic stress. In particular, a quantitative hyperbolic function model between peak–peak values and elastic stress is established, and the exponential function relationship between Bz_pp_ and plastic deformation is established, all of which can be used as a quantitative evaluation model for stress in ENDT.

(d) The magnetic flux signals decrease with the increase in plastic deformation. It is worth noting that the peak–peak values decrease greatly with the increase in the plastic deformation, and the downtrend becomes smooth for larger plastic deformation, which is caused by the evolution of dislocation morphology and domain wall pinning under plastic deformation.

To summarize, the proposed model can accurately describe the magneto–mechanical coupling effect of ferromagnetic materials under elasto–plastic deformation, and the finite element–infinite element coupling method can be effectively used for transient analysis of magnetic nondestructive testing. Meanwhile, the proposed model can be combined with simulation analysis, which is expected to be applied in the quantitative study of non-destructive testing techniques.

## Figures and Tables

**Table 1:** Comparison of NMSE between the simulation results and experimental results [30].

NMSE	FE-IFEM	LTB-FEM	MTB-FEM	STB-FEM
50 Hz	0.035	0.038	0.093	0.438
200 Hz	0.035	0.042	0.083	1.529

**Table 2:** The NMSE between the calculation results of each model and the experimental data.

Model	The Proposed Model	Sun’s Model [7]	Yang’s Model [31]
H*x*	7.96%	14.07%	9.61%
H*z*	40.26%	49.16%	48.15%

**Table 3:** Parameters for Jiles–Atherton model.

Parameter	Values on the Diagonal
Saturation magnetization	1.31 × 10^6^ A/m, 1.33 × 10^6^ A/m, 1.31 × 10^6^ A/m
Domain wall density	233.78 A/m, 177.856 A/m, 233.78 A/m
Pinning loss	374.975 A/m, 232.652 A/m, 374.975 A/m
Magnetization reversibility	0.736, 0.652, 0.736
Inter-domain coupling	5.62 × 10^−4^, 4.17 × 10^−4^, 5.62 × 10^−4^

**Table 4:** Corresponding relationship between driving voltage, hysteresis loop, and magnetic signal.

Signal	Figure Number	Range				
Driving voltage	Figure 8a	A–B	B–C	C–D	D–E	E–F
Hysteresis loop	Figure 8b	a–b	b–c	c–d	d–e	e–f
B*x*	Figure 8c	a1–b1	b1–c1	c1–d1	d1–e1	e1–f1
B1*x*	Figure 8c	a11–b11	b11–c11	c11–d11	d11–e11	e11–f11
B*z*	Figure 8d	a2–b2	b2–c2	c2–d2	d2–e2	e2–f2
B1*z*	Figure 8d	a21–b21	b21–c21	c21–d21	d21–e21	e21–f21

**Figure A1:**
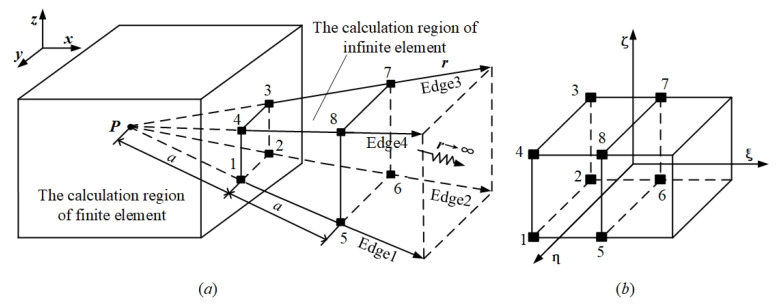
Infinite geometry mapping for 3D problems. (**a**) Sub-element, (**b**) parent element, the coordinates of point: 1 (−1,1,−1), 2 (−1,−1,−1), 3 (−1,−1,1), 4 (−1,1,1).

## Data Availability

The original contributions presented in the study are included in the article, further inquiries can be directed to the corresponding author.

## Appendix A

### Appendix A.1. Finite Element Method

According to the relationship between the magnetic field and magnetic induction density β=μH (where μ is magnetic permeability, $\mu =\mu_{0}\mu_{r},\;\mu_{0}$ is the vacuum magnetic permeability, and $\mu_{r}$ is the relative permeability), the vector potential of the magnetic field B=∇×A, and the Coulomb gauge ∇·A=0, the equation $\left\{\begin{array}{c} \nabla \times H=J_{0}\\ \nabla \cdot B=0 \end{array}\right.$ can be written as [21]

(A1) $$-\frac{1}{\mu }\nabla^{2}A=J_{0}$$

The Galerkin finite element method is used to discretize Equation (6), and the form of the weight function *N_j_* is the same as the shape function *N_i_* [21]. Therefore, in the finite element solution area Ω, Equation (A1) can be written as

(A2) $$-\int_{\Omega }\frac{1}{\mu }\nabla^{2}A\cdot N_{j}d\Omega =\int_{\Omega }J_{0}\cdot N_{j}d\Omega$$

According to Green’s theorem [21], combined with the element interpolation function of the magnetic vector potential $\hat{A}=\sum_{i\epsilon \hat{\Omega }}{\hat{A}}_{i}N_{i}$, the left-hand side of Equation (A2) can be written as

(A3) $$-\int_{\Omega }\frac{1}{\mu }\nabla^{2}A\cdot N_{j}d\Omega =\frac{1}{\mu }\sum_{i\epsilon \hat{\Omega }}s_{ij}{\hat{A}}_{i}-\frac{1}{\mu }\sum_{i\epsilon \hat{\Omega }}h_{ij}\frac{\partial {\hat{A}}_{i}}{\partial n}$$

where $s_{ij}=\int_{\hat{\Omega }}\nabla N_{i}\nabla N_{j}d\Omega ,\;h_{ij}=\int_{\hat{\Omega }}\nabla N_{i}\nabla N_{j}d\Omega \cdot \frac{1}{\mu }\sum_{i\epsilon \hat{\Omega }}h_{ij}\frac{\partial {\hat{A}}_{i}}{\partial n}$ is the boundary integral term, and its calculation depends on the boundary conditions of the finite element, thus its processing method in this paper will be explained in the finite element–infinite element coupling analysis in the section on infinite analysis.

The first term on the right-hand side of Equation (A3) can be written as

(A4) $$\frac{1}{\mu }\sum_{i\epsilon \hat{\Omega }}s_{ij}{\hat{A}}_{i}=\frac{1}{\mu }K_{ij}^{1}A_{e}$$

where $K_{ij}^{1}$ is the stiffness matrix, $K_{ij}^{1}=\int_{e}\left(\frac{\partial N_{i}}{\partial x}\;\frac{\partial N_{j}}{\partial x}+\frac{\partial N_{i}}{\partial y}\;\frac{\partial N_{j}}{\partial y}+\frac{\partial N_{i}}{\partial z}\;\frac{\partial N_{j}}{\partial z}\right)$, $A_{e}=\left(A_{1},A_{2},\ldots ,A_{i}{)}^{T}\right.$.

According to the transformation relationship between global coordinates and local coordinates [22],

(A5) $$\left\{\begin{array}{c} \frac{\partial N_{i}}{\partial \xi }\\ \frac{\partial N_{i}}{\partial \eta }\\ \frac{\partial N_{i}}{\partial \varsigma } \end{array}\right\}=\left[\begin{array}{c} \frac{\partial x}{\partial \xi }\\ \frac{\partial x}{\partial \eta }\\ \frac{\partial x}{\partial \varsigma } \end{array}\begin{array}{c} \frac{\partial y}{\partial \xi }\\ \frac{\partial y}{\partial \eta }\\ \frac{\partial y}{\partial \varsigma } \end{array}\begin{array}{c} \frac{\partial z}{\partial \xi }\\ \frac{\partial z}{\partial \eta }\\ \frac{\partial z}{\partial \varsigma } \end{array}\right]\left\{\begin{array}{c} \frac{\partial N_{i}}{\partial x}\\ \frac{\partial N_{i}}{\partial y}\\ \frac{\partial N_{i}}{\partial z} \end{array}\right\}=\left[J\right]\left\{\begin{array}{c} \frac{\partial N_{i}}{\partial x}\\ \frac{\partial N_{i}}{\partial y}\\ \frac{\partial N_{i}}{\partial z} \end{array}\right\}$$

(A6) $$d\Omega =dxdydz=\left[J\right]d\xi d\eta d\varsigma$$

where $\left[J\right]$ is the Jacobian matrix.

(A7) $$\left[J\right]=\left[\begin{array}{c} \frac{\partial x}{\partial \xi }\\ \frac{\partial x}{\partial \eta }\\ \frac{\partial x}{\partial \varsigma } \end{array}\;\begin{array}{c} \frac{\partial y}{\partial \xi }\\ \frac{\partial y}{\partial \eta }\\ \frac{\partial y}{\partial \varsigma } \end{array}\;\begin{array}{c} \frac{\partial z}{\partial \xi }\\ \frac{\partial z}{\partial \eta }\\ \frac{\partial z}{\partial \varsigma } \end{array}\right]=\left[\begin{array}{c} \sum \frac{\partial N_{i}}{\partial \xi }x_{i}\\ \sum \frac{\partial N_{i}}{\partial \eta }x_{i}\\ \sum \frac{\partial N_{i}}{\partial \varsigma }x_{i} \end{array}\;\begin{array}{c} \sum \frac{\partial N_{i}}{\partial \xi }y_{i}\\ \sum \frac{\partial N_{i}}{\partial \eta }y_{i}\\ \sum \frac{\partial N_{i}}{\partial \varsigma }y_{i} \end{array}\;\begin{array}{c} \sum \frac{\partial N_{i}}{\partial \xi }z_{i}\\ \sum \frac{\partial N_{i}}{\partial \eta }z_{i}\\ \sum \frac{\partial N_{i}}{\partial \varsigma }z_{i} \end{array}\right]$$

According to Equation (A5), $\;\frac{\partial N_{i}}{\partial x}\;,\;\frac{\partial N_{i}}{\partial y}\;,\;\frac{\partial N_{i}}{\partial z}$ can be expressed as a local coordinate:

$$\left\{\begin{array}{c} \frac{\partial N_{i}}{\partial x}\\ \frac{\partial N_{i}}{\partial y}\\ \frac{\partial N_{i}}{\partial z} \end{array}\right\}=[J{]}^{-1}\left\{\begin{array}{c} \frac{\partial N_{i}}{\partial \xi }\\ \frac{\partial N_{i}}{\partial \eta }\\ \frac{\partial N_{i}}{\partial \varsigma } \end{array}\right\}.$$

Let $\frac{\partial N_{i}}{\partial x}=F_{ix}\left(\xi ,\eta ,\varsigma \right)\;,\;\frac{\partial N_{i}}{\partial y}=F_{iy}\left(\xi ,\eta ,\varsigma \right),\;and\;\frac{\partial N_{i}}{\partial z}=F_{iz}\left(\xi ,\eta ,\varsigma \right)$, then the stiffness matrix can be written as $K_{ij}^{1}=\int_{-1}^{1}\int_{-1}^{1}\int_{-1}^{1}\left[F_{ix}F_{jx}+F_{ix}F_{jx}+F_{ix}F_{jx}\right]\left[J\right]d\xi d\eta d\varsigma$.

### Appendix A.2. Infinite Element Method

The principle of the infinite element method is to propagate the magnetic field to infinity by coordinate mapping, thereby reducing the simulation error caused by the finite air region. Among the many infinite element mapping methods, the Astley mapping infinite element method [18,19] deals with transient problems very simply. Thus, the Astley infinite element method is used as the boundary condition of the finite element in this paper.

The infinite element coordinate mapping of the 3D model is shown in Figure A1. The infinite elements have directionalities that take the coordinate origin P as the only starting point of the mapping and map to infinity through the boundary nodes 1–4 of the finite elements. In Figure A1a, nodes 1–8 are the basic elements of the infinite element, and nodes 1–4 are nodes on a certain element on the finite element boundary. The local coordinate system of the infinite element is defined with the ξ, η, and ς  coordinate axes, as shown in Figure A1b, and the local coordinate of the η, and   ς axes take values between −1 and 1. By contrast, the direction of ξ points from the point P to infinity, with the value ξ=1 on the interface boundary, and taking the range of 1 to ∞. Thus, any point in the infinite element can be expressed as

(A8) $$\left\{\begin{array}{c} x=\sum_{i=1}^{8}M_{i}\left(\xi ,\eta ,\varsigma \right)x_{i}\;\\ y=\sum_{i=1}^{8}M_{i}\left(\xi ,\eta ,\varsigma \right)y_{i}\\ z=\sum_{i=1}^{8}M_{i}\left(\xi ,\eta ,\varsigma \right)z_{i} \end{array}\right.$$

where $x_{i\;},\;\;y_{i}\;,\;\;and\;z_{i}$ are the spatial coordinates of infinite element nodes and $M_{i}\left(\xi ,\eta ,\varsigma \right)\;$ is the mapping function, which can be expressed as

(A9) $$\left\{\begin{array}{c} M_{1}=-\frac{\xi \left(1+\eta \right)\left(1-\varsigma \right)}{2\left(1-\xi \right)}\\ M_{2}=-\frac{\xi \left(1+\eta \right)\left(1-\varsigma \right)}{2\left(1-\xi \right)}\\ M_{3}=-\frac{\xi \left(1+\eta \right)\left(1-\varsigma \right)}{2\left(1-\xi \right)}\\ M_{4}=-\frac{\xi \left(1+\eta \right)\left(1-\varsigma \right)}{2\left(1-\xi \right)} \end{array}\;,\;\left\{\begin{array}{c} M_{5}=-\frac{\left(1+\xi \right)\left(1-\varsigma \right)\left(1-\varsigma \right)}{4\left(1-\xi \right)}\\ M_{6}=-\frac{\left(1+\xi \right)\left(1-\varsigma \right)\left(1-\varsigma \right)}{4\left(1-\xi \right)}\\ M_{7}=-\frac{\left(1+\xi \right)\left(1-\varsigma \right)\left(1-\varsigma \right)}{4\left(1-\xi \right)}\\ M_{8}=-\frac{\left(1+\xi \right)\left(1-\varsigma \right)\left(1-\varsigma \right)}{4\left(1-\xi \right)} \end{array}\right.\;\;\right.$$

And the magnetic potential satisfies

(A10) $$A=\sum_{i=1}^{8}N_{i}\left(\xi ,\eta ,\varsigma \right)A_{i}$$

where $N_{i}$ is the shape function, which can be obtained by modifying the quadratic Lagrangian interpolation function with the coefficient $\frac{1-\xi }{2}$ [18,19]:

(A11) $$\left\{\begin{array}{c} N_{1}=-\frac{\xi \left(1-\xi {)}^{2}\left(1+\eta \right)\left(1-\varsigma \right)\right.}{16}\\ N_{2}=-\frac{\xi \left(1-\xi {)}^{2}\left(1+\eta \right)\left(1-\varsigma \right)\right.}{16}\\ N_{3}=-\frac{\xi \left(1-\xi {)}^{2}\left(1+\eta \right)\left(1-\varsigma \right)\right.}{16}\\ N_{4}=-\frac{\xi \left(1-\xi {)}^{2}\left(1+\eta \right)\left(1-\varsigma \right)\right.}{16} \end{array}\;,\;\left\{\begin{array}{c} N_{5}=-\frac{\left(1-\xi^{2})\left(1+\xi \right)\left(1+\eta \right)\left(1-\varsigma \right)\right.}{8}\\ N_{6}=-\frac{\left(1-\xi^{2})\left(1-\xi \right)\left(1+\eta \right)\left(1-\varsigma \right)\right.}{8}\\ N_{7}=-\frac{\left(1-\xi^{2})\left(1-\xi \right)\left(1-\eta \right)\left(1+\varsigma \right)\right.}{8}\\ N_{8}=-\frac{\left(1-\xi^{2})\left(1-\xi \right)\left(1+\eta \right)\left(1+\varsigma \right)\right.}{8} \end{array}\right.\;\;\right.$$

In infinite element analysis, the Jacobi matrix in infinite elements $\;J^{\Theta }$ is calculated by mapping functions. It can be expressed as

(A12) $$\left[J^{\Theta }=\left[\begin{array}{c} \frac{\partial x}{\partial \xi }\\ \frac{\partial x}{\partial \eta }\\ \frac{\partial x}{\partial \varsigma } \end{array}\begin{array}{c} \;\frac{\partial y}{\partial \xi \;}\;\\ \frac{\partial y}{\partial \eta }\\ \frac{\partial y}{\partial \varsigma } \end{array}\begin{array}{c} \frac{\partial z}{\partial \xi }\\ \frac{\partial z}{\partial \eta }\\ \frac{\partial z}{\partial \varsigma } \end{array}\right]=\left[\begin{array}{c} \sum_{i=1}^{8}\frac{\partial M_{i}}{\partial \xi }x_{i}\\ \sum_{i=1}^{8}\frac{\partial M_{i}}{\partial \eta }x_{i}\\ \sum_{i=1}^{8}\frac{\partial M_{i}}{\partial \varsigma }x_{i} \end{array}\;\;\;\begin{array}{c} \sum_{i=1}^{8}\frac{\partial M_{i}}{\partial \xi }y_{i}\\ \sum_{i=1}^{8}\frac{\partial M_{i}}{\partial \eta }y_{i}\\ \sum_{i=1}^{8}\frac{\partial M_{i}}{\partial \varsigma }y_{i} \end{array}\;\;\;\begin{array}{c} \sum_{i=1}^{8}\frac{\partial M_{i}}{\partial \xi }z_{i}\\ \sum_{i=1}^{8}\frac{\partial M_{i}}{\partial \eta }z_{i}\\ \sum_{i=1}^{8}\frac{\partial M_{i}}{\partial \varsigma }z_{i} \end{array}\right]\;\right]$$

According to the analysis of the finite element method in Appendix A.1, the stiffness matrix of an infinite element can be expressed as

(A13) $$K_{ij}^{2}=\int_{-1}^{1}\int_{-1}^{1}\int_{-1}^{1}\left[F_{ix}F_{jx}+F_{iy}F_{jy}+F_{iz}F_{jz}\right]\left[J\right]d\xi d\eta d\varsigma$$

where $i,j=1,\ldots ,8,\;and\;K_{ij}^{2}$ is the stiffness value of the infinite element.

### Appendix A.3. Finite Element–Infinite Element Coupling

The magnetic potential extends to infinity through the infinite element and decays to zero at infinity. Thus, there is no need to consider the boundary conditions of the magnetic field, that is, the boundary integral in Equation (A3) does not need to be considered in the finite element calculation. Meanwhile, in a simulation process, to fit the complex specimen better, the hexahedral finite element of the specimen can be subdivided into tetrahedral finite elements, and the total nodes number of tetrahedral finite elements of the specimen is the same as that of the original hexahedral nodes to facilitate coupling with the infinite element hexahedron [19]. Further, the finite element–infinite element coupling process is only necessary to add the stiffness matrix values calculated by finite elements $K_{ij}^{1}$ and infinite elements $K_{ij}^{2}$, respectively, to the corresponding position of the total stiffness matrix according to node numbers, which realizes the effective coupling of two elements and two methods.

(A14) $$\left[\begin{array}{ccc} \ldots & \ldots & \ldots \\ \ldots & K_{ij} & \ldots \\ \ldots & \ldots & \ldots \end{array}\right]=\left[\begin{array}{ccc} \ldots & \ldots & \ldots \\ \ldots & K_{ij}^{1} & \ldots \\ \ldots & \ldots & \ldots \end{array}\right]+\left[\begin{array}{ccc} \ldots & \ldots & \ldots \\ \ldots & K_{ij}^{2} & \ldots \\ \ldots & \ldots & \ldots \end{array}\right]$$

## Appendix B

### Appendix B.1. The Magneto–Mechanical Coupling Model

According to the law of thermodynamics and the relationship between heat energy and work carried out in the magnetization process of soft magnetic materials [2,3,4,5,6], when the molecular coupling energy 12μ0αMan2, demagnetizing energy 12μ0NdMan2, and stress equivalent magnetic field are taken into account, the energy per unit volume of material is [3,4,5,6]

(A15) $$dU=TdS+\mu_{0}\left(H+\alpha M_{an}-N_{d}M_{an}+H_{\sigma }\right)dM_{an}+\sigma d\varepsilon$$

where *S* is volume entropy, T is the ambient temperature, α is the Weiss coupling coefficient, $N_{d}$ is the demagnetization coefficient, $N_{d}=N_{s}+N_{m},\;\;N_{s}$ is the geometrical demagnetization factor related to the material geometry, $M_{m}$ is the demagnetization factor of internal defects in materials, and *M_an_* is the anhysteresis magnetization.

The effective field equation is introduced: $H_{eff}=H+\left(\alpha -N_{d}\right)M_{an}+H_{\sigma }$. Equation (A15) can be written as

(A16) $$dU=TdS+\mu_{0}H_{eff}dM_{an}+\sigma d\varepsilon$$

According to the Gibbs free energy equation dG=dU−TdS−SdT−εdσ−σdε [2,3,4], when the temperature change is not considered, namely, dT=0

(A17) $$\left\{\begin{array}{c} \varepsilon \left(\sigma ,T,M_{an}\right)=-\frac{\partial G}{\partial \sigma }\\ H_{eff}=\frac{1}{\mu_{0}}\frac{\partial G}{\partial M_{an}} \end{array}\right.$$

Let *G*(*σ*,*M_an_*) be Taylor expanded at the point (*σ*,*M_an_*) = (0,0), the elastic Gibbs free energy *G*(0,0) is a constant, that is to say, the derivative is 0. In the natural state, we know that *ε* = 0, *H* = 0 when *σ* = 0, *M_an_* = 0. According to the thermodynamic equation, the following results can be obtained: $\partial G\left(0,0\right)/\partial \sigma =0,\;\partial G\left(0,0\right)/\partial M_{an}=0$. According to the relationship between magnetic quantities and hysteresis loops, it can be seen that the functional relationship between magnetic field strength and magnetization always conforms to the odd function relationship, that is to say, the even term of magnetization intensity in $H_{eff}\left(\sigma ,M_{an}\right)=1/\mu_{0}\cdot \partial G/\partial M_{an}$ relation can be ignored, and the odd number term can be reserved, corresponding to the Gibbs expansion. It can be seen that the odd number term of magnetization intensity can be ignored and only the even term can be retained.

The influence of the higher-order term of magnetization on the Magnetostriction is very small, which can almost be ignored. Therefore, only *M_an_* quadratic terms are retained in the coupling term. Therefore, we can get the following results:

(A18) $$\begin{array}{c} \varepsilon \left(\sigma ,M_{an}\right)=-\sigma \frac{\partial^{2}G\left(0,0\right)}{\partial \sigma^{2}}-\frac{1}{2!}\sigma^{2}\frac{\partial^{3}G\left(0,0\right)}{\partial \sigma^{3}}-\frac{1}{3!}\sigma^{3}\frac{\partial^{4}G\left(0,0\right)}{\partial \sigma^{4}}-\ldots -\frac{1}{2!}\\ \;\;\;\;\;\;\;\cdot \left(\frac{\partial^{3}G\left(0,0\right)}{\partial \sigma \partial M_{an}^{2}}+\sigma \frac{\partial^{4}G\left(0,0\right)}{\partial \sigma^{2}\partial M_{an}^{2}}+\frac{1}{2}\sigma^{2}\frac{\partial^{5}G\left(0,0\right)}{\partial \sigma^{3}\partial M_{an}^{2}}+\ldots \right)M_{an}^{2} \end{array}$$

(A19) $$\begin{array}{c} H_{eff}=M_{an}\frac{\partial^{2}G\left(0,0\right)}{\partial M_{an}^{2}}+\frac{1}{3!}M_{an}^{3}\frac{\partial^{4}G\left(0,0\right)}{\partial M_{an}^{4}}+\frac{1}{5!}M_{an}^{5}\frac{\partial^{6}G\left(0,0\right)}{\partial M_{an}^{6}}+\cdots \\ \;\;\;\;\;\;\;\;\;\;\;+\left(\sigma \frac{\partial^{3}G\left(0,0\right)}{\partial \sigma \partial M_{an}^{2}}+\frac{1}{2}\sigma^{2}\frac{\partial^{4}G\left(0,0\right)}{\partial \sigma^{2}\partial M_{an}^{2}}+\frac{1}{6}\sigma^{2}\frac{\partial^{5}G\left(0,0\right)}{\partial \sigma^{3}\partial M_{an}^{2}}+\ldots \right)M_{an} \end{array}$$

The above two formulas are magneto–elastic coupling constitutive relations. The expression without magnetization *M_an_* in the above-mentioned strain equation is mainly the elastic strain produced by the action of stress *σ*, that is, the elastic deformation under the prestressing. The representative magnetostrictive strain related to the magnetization *M_an_*, which is generated when the magnetic field is applied under the prestress, can be used as the magnetostrictive strain *λ*(*σ*, *M_an_*). According to the previous research, it can be seen that the magnetostrictive strain and the magnetization are non-linear relations. According to the theory of metal elastic deformation, the strain only under the action of stress is mainly composed of two parts: one is linear with stress, the other is nonlinear with stress. Therefore, the strain caused by prestressing in ferromagnetic materials can be divided into two parts: the strain caused by magnetic domain wall displacement and the part independent of domain wall displacement. The relationship between strain and stress caused by domain wall movement is linear, expressed by *σ*/*E_s_*, where *E_s_* represents Young’s modulus of the material, while the other part is nonlinear and expressed by a nonlinear function *λ*_0_(*σ*). That is, the part of Equation (9) only related to stress can be simplified as follows:

(A20) $$-\sigma \frac{\partial^{2}G\left(0,0\right)}{\partial \sigma^{2}}-\frac{1}{2!}\sigma^{2}\frac{\partial^{3}G\left(0,0\right)}{\partial \sigma^{3}}..-\frac{1}{n!}\sigma^{n}\frac{\partial^{n+1}G\left(0,0\right)}{\partial \sigma^{n+1}}=\frac{\sigma }{E_{s}}+\lambda_{0}\left(\sigma \right)$$

According to the analysis of the magnetization process, we know that when the magnetostriction reaches the maximum value, the maximum magnetic field intensity required for the wall shift is achieved [3]. When the prestress is 0, the corresponding magnetization is the saturated wall shift magnetization *M_ws_* and the stress-related saturation magnetization *M*_0_(*σ*). The magnetostrictive strain increases monotonously with the magnetization from the application of the magnetic field to the saturation wall shift magnetization. It can be seen from the analysis that only $M_{an}^{2}$ plays a role in this process, then, with the increase in the magnetic field, the magnetostrictive strain decreases gradually until saturation due to the rotation of the domain wall [2,3,4]. During the magnetization process, the magnetostrictive strain is the extreme point at the maximum magnetostriction and then decreases monotonously until saturation. At this time, the effect of $M_{an}^{4}$ on the strain should be considered. Since *M_ws_* is the extreme value, it is taken as the dividing point.

The magnetostrictive strain *λ*(*σ*,*M_an_*) obtained by applying magnetic field H under prestressing force takes the deformation produced by prestressing as the starting point of measurement. Therefore, *λ*(*σ*,*M_an_*) is always 0 before magnetization, and *λ*(*σ*,*M_an_*) reaches the maximum value *λ*_max_(*σ*) when magnetization reaches saturation wall shift magnetization. Then, with the increase in magnetization, the domain wall begins to rotate and the magnetostrictive strain begins to decrease. Therefore, the maximum magnetostrictive strain *λ*_max_(*σ*) should be equal to the difference between the maximum magnetostrictive strain *λ_s_*(*σ*) in the free state and the nonlinear part $\lambda_{0}\left(\sigma \right)=\frac{1}{4}\lambda_{s}\left[1+3\tanh\left(\frac{\beta \sigma }{\sigma_{s}}-arctan\;h\left(\frac{1}{3}\right)\right)\right]\cdot$

(A21) $$\lambda_{max}\left(\sigma \right)=\lambda_{s}-\lambda_{0}\left(\sigma \right).$$

Considering that the corresponding part of saturation wall shift magnetization *M*_0_(*σ*), it can be seen that *M_an_*-*M*_0_ works only after the cut-off point, and it does not work in the first half. Therefore, it is necessary to introduce a step function correction to the fourth power [4].

(A22) $$\vartheta =\left\{\begin{array}{ll} \theta , & M\geq M_{0}\left(\sigma \right)\\ 0, & M<M_{0}\left(\sigma \right) \end{array}\right.$$

The terms related to the second power of magnetization in the corresponding constitutive equation can be simplified as follows:

(A23) $$-\frac{1}{2!}\cdot \left(\frac{\partial^{3}G\left(0,0\right)}{\partial \sigma \partial M_{an}^{2}}+\sigma \frac{\partial^{4}G\left(0,0\right)}{\partial \sigma^{2}\partial M_{an}^{2}}+\frac{1}{2}\sigma^{2}\frac{\partial^{5}G\left(0,0\right)}{\partial \sigma^{3}M_{an}^{2}}+\ldots \right)M_{an}^{2}=\frac{\lambda_{s}-\lambda_{0}}{M_{an}^{2}}M_{an}^{2}-\vartheta \frac{\lambda_{s}k\left(1-\tau \right)\sigma }{\left(M_{s}-M_{0}{)}^{2}\right.}\left(M_{an}-M_{0}{)}^{2}\right.$$

So, the total strain can be written as

(A24) $$\varepsilon \left(\sigma ,M_{an}\right)=\frac{\sigma }{E_{s}}+\lambda_{0}\left(\sigma \right)+\frac{\lambda_{max}\left(\sigma \right)}{M_{ws}^{2}}M_{an}^{2}-\vartheta \frac{\lambda_{s}k\left(1-\tau \right)}{\left(M_{s}-M_{0}{)}^{2}\right.}$$

The effective field can be written as

(A25) $$\begin{array}{ll} H_{eff}\left(\sigma ,M_{an}\right) & =H+\alpha M_{an}-N_{d}M_{an}+\frac{2\lambda_{max}\left(\sigma \right)}{\mu_{0}M_{ws}^{2}}M_{an}-\frac{2\vartheta \lambda_{s}k\left(1-\tau \right)}{\left(M_{s}-M_{0}{)}^{2}\right.}\;M_{an}\\ & \;=\xi M_{an}+\frac{2\left[\lambda_{max}\sigma -\Lambda \left(\sigma \right)\right]}{\mu_{0}M_{ws}^{2}}\;M_{an}-\frac{2\vartheta \lambda_{s}k\left(1-\tau \right)\sigma }{\left(M_{s}-M_{0}{)}^{2}\right.}\;M_{an} \end{array}$$

The equivalent magnetic field strength under actual magnetization *M* can be obtained by replacing *M_an_* with *M* during actual magnetization [24].

Therefore, the effective fields can be written as

(A26) $$H_{e}=\xi M+\frac{2\left[\lambda_{s}\sigma -\Lambda \left(\sigma \right)\right]}{\mu_{0}M_{ws}^{2}}M-\frac{2\vartheta \lambda_{s}k\left(1-\tau \right)\sigma }{\left(M_{s}-M_{0}{)}^{2}\right.}$$
